# Subjective Memory Complaints and Objective Memory: The Role of Depressive Symptoms and Health Insurance Coverage

**DOI:** 10.1093/geroni/igaf122.3237

**Published:** 2025-12-31

**Authors:** Hyun Moon, Jordana Breton, Elizabeth Muñoz

**Affiliations:** The University of Texas at Austin, Austin, Texas, United States; The University of Texas at Austin, Austin, Texas, United States; University of Texas at Austin, Austin, Texas, United States

## Abstract

Research indicates that higher subjective memory complaints (SMC) prospectively predict memory performance, with depressive symptoms mediating this process. We examined access to health insurance coverage as a marker of access to and availability of timely healthcare as a moderator of this indirect association. Using two-wave data from the Health and Aging Brain Study – Health Disparities, we expected to replicate the indirect effect reported in the literature from SMC (Time 1) to memory performance (Time 2) via depressive symptoms (Time 2). We expanded on this by testing the hypothesis that this mediation effect would be stronger for those without health insurance coverage compared to those with health insurance. We constructed a variable for memory performance, and fitted a multigroup structural equation model with 1,000 bootstrap samples to test group differences. Covariate adjusted models showed that higher SMC at Time 1 predicted greater depressive symptoms at Time 2, which were associated with lower memory performance at Time 2 (b = -0.027, p = .005, 95% CI [-0.045, -0.009]). Contrary to our expectation, a significant group difference (χ2(2) = 11.112, p = .004) indicated that this indirect effect was observed only among individuals with health insurance coverage (b = -0.026, p = .016, 95% CI [-0.049, -0.005]). These findings suggest that the role of SMC on memory performance may be stronger among those with health insurance coverage. Future research should explore mechanisms underlying this phenomenon (e.g., ongoing monitoring of memory concerns leading to greater depressive symptoms) to inform practical applications.

